# Validation of microarray data in human lymphoblasts shows a role of the ubiquitin-proteasome system and NF-*k*B in the pathogenesis of Down syndrome

**DOI:** 10.1186/1755-8794-6-24

**Published:** 2013-07-05

**Authors:** Barbara Granese, Iris Scala, Carmen Spatuzza, Anna Valentino, Marcella Coletta, Rosa Anna Vacca, Pasquale De Luca, Generoso Andria

**Affiliations:** 1 Department of Pediatrics, Federico II University, Naples 80131, Italy; 2 Department of Biotechnological Sciences, Federico II University, Naples 80131, Italy; 3 Institute of Biomembranes and Bioenergetics, National Council of Research, Bari 70126, Italy; 4 Stazione Zoologica “A. Dohrn”, c/o BioGeM, Via Camporeale, Ariano Irpino 83031, Italy

**Keywords:** Down syndrome, Trisomy 21, Expression, Ubiquitin-proteasome system, NF-*k*B

## Abstract

**Background:**

Down syndrome (DS) is a complex disorder caused by the trisomy of either the entire, or a critical region of chromosome 21 (21q22.1-22.3). Despite representing the most common cause of mental retardation, the molecular bases of the syndrome are still largely unknown.

**Methods:**

To better understand the pathogenesis of DS, we analyzed the genome-wide transcription profiles of lymphoblastoid cell lines (LCLs) from six DS and six euploid individuals and investigated differential gene expression and pathway deregulation associated with trisomy 21. Connectivity map and PASS-assisted exploration were used to identify compounds whose molecular signatures counteracted those of DS lymphoblasts and to predict their therapeutic potential. An experimental validation in DS LCLs and fetal fibroblasts was performed for the most deregulated GO categories, i.e. the ubiquitin mediated proteolysis and the NF-*k*B cascade.

**Results:**

We show, for the first time, that the level of protein ubiquitination is reduced in human DS cell lines and that proteasome activity is increased in both basal conditions and oxidative microenvironment. We also provide the first evidence that NF-*k*B transcription levels, a paradigm of gene expression control by ubiquitin-mediated degradation, is impaired in DS due to reduced I*k*B-alfa ubiquitination, increased NF-*k*B inhibitor (I*k*B-alfa) and reduced p65 nuclear fraction. Finally, the DSCR1/DYRK1A/NFAT genes were analysed. In human DS LCLs, we confirmed the presence of increased protein levels of DSCR1 and DYRK1A, and showed that the levels of the transcription factor NFATc2 were decreased in DS along with a reduction of its nuclear translocation upon induction of calcium fluxes.

**Conclusions:**

The present work offers new perspectives to better understand the pathogenesis of DS and suggests a rationale for innovative approaches to treat some pathological conditions associated to DS.

## Background

Down syndrome (DS) (MIM 190685) is a human complex disorder caused by the trisomy of either the entire, or a critical region of chromosome (chr) 21 (21q22.1-22.3). DS phenotypes are often variable. Intellectual disability and hypotonia are the hallmarks of the syndrome, while a wealth of distinct clinical manifestations, including congenital malformations, increased incidence of cancer, immune and endocrine abnormalities occur only in subsets of DS subjects. DS is also characterized by premature aging and dementia with neurological features that mimic those found in Alzheimer’s disease. The underlying molecular mechanisms of DS are largely unknown. Several genome-wide expression studies have been performed both in mouse and human trisomic tissues. While DS mice models have unravelled a generalized overexpression of triplicated genes [1-4], the analysis of human DS tissues showed contradictory results. In fact, some studies reported the selective over-expression of a limited subset of chr 21 genes [5-9], and others described subtle upregulations of chr 21 genes associated to a secondary, generalized and more extreme transcriptional deregulation of genes mapping on other chromosomes [10-15]. An additional level of complexity comes from the observation that gene expression differs extensively among unaffected individuals [16-19], including the expression of a number of chr 21 genes [20,21]. Therefore, some authors suggested to regard as poor candidates for DS pathogenesis those genes with high expression variation among controls and as reliable candidates those genes over-expressed in DS and tightly regulated in euploid cells [21-23]. Gene expression studies failed to provide definitive results; however, evidence in human DS cells point to the presence of abnormalities of extracellular matrix, of mitochondrial function and other metabolic pathways, including purine metabolism, in fetal specimens [5,11,13], and changes in transcriptional regulation, oxidative stress and immune-related genes in adult tissues [6,12,14,24,25]. Recently, the effect of single chr 21 genes on the trisomic trancriptome was established by comparing the genome-wide expression from mouse ES cells, engineered to host the whole human chr 21, with those overexpressing only single chr 21 genes. A subset of genes, including Runx1, Erg, Nrip, Olig2, PdxK and Aire, produced the strongest transcriptional response when overexpressed [26]. More recently, Vilardell et al. [27] performed a meta-analysis from 45 publicly available DS data sets, from both human and mouse transcriptome and proteome. The identified biological functions were mainly related to nervous system development, neurodegenerative disorders (e.g. Huntington’s disease, Alzheimer’s disease and Parkinson’s disease) and defects in synapsis function (e.g. axon guidance, NGF signalling). Seventy distinct transcription factors, including RelA, NFATc1, NFATc2 and NFATc3, were identified as being affected by dosage imbalance.

Besides genome-wide expression analysis, few studies attempted to identify selected gene networks associated to specific DS features and to characterize molecular and biochemical functions disrupted in DS. In mice, Arron et al. [28] found that two critical chr 21 genes (DYRK1A and DSCR1, also known as RCAN1) act synergistically to control the nuclear localization of NFAT family of transcription factors and that knock-out mice for NFATc1, NFATc2, NFATc3 and NFATc4 display cardiovascular, neurological, skeletal and immune phenotypes strikingly similar to DS. In addition, DSCR1 promotes neurotoxicity [29] and attenuates the inflammatory response by stabilizing I*k*B-alfa [30]. The extra copy of DYRK1A in DS has been also associated to early onset of Alzheimer’s disease [31] and to defective neuronal development mediated by the reduction of REST, a key regulator of pluripotency and neuronal differentiation [32]. DSCR1/NFAT pathway was also associated with neuronal susceptibility to oxidative stress [33], a biochemical feature of DS. Finally, a limited number of functional studies in human DS cells have unravelled a disruption of mitochondrial function as a pathogenetic trigger [34-37].

In this study we analyzed the genome-wide transcription profile of lymphoblastoid cell lines (LCLs) from DS and control subjects to investigate differential gene expression and pathway deregulation associated with trisomy 21. This cellular model has been widely used to analyze the expression profiles of chr 21 genes [21,23,38] as well as of other diseases including cancer [39-41] and neurodegenerative disorders [42,43]. Diseased versus control and intra-group comparisons were used to analyze the gene expression levels of chr 21 genes. The experimental validation focused on the most deregulated gene ontology (GO) categories to confirm their imbalance in DS, i.e. the ubiquitin mediated proteolysis and the NF-*k*B cascade. Interestingly, these two celullar processes are closely interconnected since I*k*B-alfa, the key modulator of NF-*k*B, can be regarded as a model protein of regulation of signal transduction by the ubiquitin-proteasome system (UPS). Moreover, both the ubiquitin mediated proteolysis and the I*k*B/NF-*k*B signal transduction interact with and regulate DSCR1 [44]. In mice, DSCR1 and DYRK1A act synergistically to prevent the nuclear occupancy of NFAT [28], a family of transcription factors activated by calcium fluxes. Finally, DSCR1 is ubiquitinated and degraded by both the proteasome and the autophagy-lysosome pathways [45]. As these pathways are interconnected and individually can cause immune response derangement, neurodegenerative diseases, premature aging and delay of cell growth, all main features of DS, our experimental validation focused on UPS, NF-*k*B and DYRK1A/DSCR1/NFAT genes. In human LCLs and fetal fibroblasts, we provide the first evidence that the level of protein ubiquitination is reduced in DS and that proteasome activity is increased in both basal conditions and oxidative microenvironment. In addition, we first show that the NF-*k*B transcriptional activity is impaired in DS due to reduced I*k*B-alfa ubiquitination, increased NF-*k*B inhibitor (I*k*B-alfa) cytosolic levels and reduced p65 nuclear fraction. Finally, the investigation of DSCR1/DYRK1A/NFAT genes, linked to the pathogenesis of DS in animal studies, confirmed increased protein levels of DSCR1 and DYRK1A and showed reduced levels of NFATc2 and decreased NFATc2 nuclear translocation upon calcium flows induction, adding new evidence to a transcriptional regulation deficit in DS.

## Methods

### Cell culture

LCLs were generated from peripheral blood samples of six karyotypically confirmed full trisomy 21 children and six age-matched controls (5 ± 1 year-old) recruited at the Department of Pediatrics, Federico II University of Naples. The study was performed in accordance with the principles of the Helsinki Declaration and was approved by the Federico II University Ethics Committee ‘Carlo Romano’. The parents of all subjects gave written informed consent before participation. Lymphocytes were isolated by ficoll-hypaque (Gibco) and resuspended in 17% FBS-RPMI-1640 and equal volume of supernatant from overgrown B95.8 cell cultures in presence of 10 μg/ml of cyclosporine (Sandimmune Oral Suspension). Cells were grown in 17% FBS-RPMI-1640 (Cambrex) supplemented with 5000 U/ml penicillin-streptomycin, 2 mM glutamine (Gibco) and 0.16 mg/ml gentamicin, in a 37°C, 5% CO_2_ incubator.

Fibroblasts from two DS and two control fetuses, spontaneously aborted at a gestational age between 14 and 19 weeks, were obtained from the Galliera Genetic Bank (Galliera Hospitals, Genova, Italy) in agreement with ethical guidelines stated in the TGB Network Charter and upon written informed consent. Cells were cultured at 37°C in D-MEM medium (Gibco) supplemented with 17% FBS, 5000U/ml penicillin-streptomycin, 2 mM glutamine (Gibco), in a 37°C, 5% CO_2_ incubator. For functional analyses, sub-confluent cultures with comparable number of culture passages (5–15) and growth rate were used. Each aliquot of LCLs was maintained in culture for no longer than 3 months (less than 160 ‘population doubling levels’) [46]. To exclude the possibility of chromosomal rearrangements (other than full trisomy 21) during culturing, LCLs were karyotyped before microarray analysis and before each experimental validation.

### Cell treatment

Calcium flows were induced with 10 ng/ml PMA and 1 mg/ml Ionomycin (Sigma) for 4 hours at 37°C. For the proteasome blocking, cells were treated with 40 μM MG132 (Calbiochem) for 6 hours at 37°C for western blot experiments and 100 μM MG-132 for 2 hours for proteasome assay. Oxidative stress was induced with 0.1 mM H_2_O_2_ for 30 minutes and recoveries were observed 1, 2 and 4 hours after cell refreshing with RPMI without H_2_O_2_. Phospho-I*k*B-alfa was analyzed after cell treatment with 40 μM MG132 for 30 minutes and incubation with 0.3 μM Calyculin A for an additional 30 minutes [47].

### RNA extraction and microarray hybridization procedure

RNAs from six DS and six control LCLs were independently hybridized on the Affymetrix HU133 plus 2.0 oligonucleotide array (Affymetrix, Santa Clara, CA), which allows the analysis of over 47,000 human transcripts including 38,500 well-characterized human genes. Total RNAs were obtained using TRIzol reagent (Gibco/BRL Life Technologies, Inc., Gaithersburg, MD) and used to prepare biotinylated target cRNA, according to the Affymetrix procedures. Quality and amount of starting RNA were confirmed using spectrophotometry and agarose gel electrophoresis. Purification of PolyA + mRNA from total RNA was performed with the Oligotex mRNA Kit (QIAGEN GmbH, Hilden, Germany): 1 μg of mRNA was used to generate first-strand cDNA by using a T7-linked oligo(dT) primer; after second strand synthesis, in-vitro transcription was performed with biotinylated UTP and CTP using the Enzo BioArray High Yield RNA Transcript Labeling Kit (Enzo Diagnostics, Farmingdale, NY). The target cRNA generated from each sample was processed according to the manufacturer’s procedures. Fragmentation of biotinylated cRNA, washing and staining were done according to the instructions provided by Affymetrix.

### Experimental design

The experimental design was a diseased versus control comparison. To assess the expression variation of chr 21 genes among DS and control samples, we focused our analysis on the variability of all chr 21 transcripts present on the array with FC ≥ 1.2 (n = 167), irrespective of the statistical analysis that excludes *‘a priori’* variable genes. Gene variability was assessed by the CV, calculated as the ratio between standard deviation and mean expression levels for each gene among samples [22]. Arbitrary cut-offs were set at 0.2 and 0.5 and transcripts were divided into three classes: CV ≤ 0.2, corresponding to the tightly regulated genes; 0.2 < CV < 0.5, corresponding to genes with little variation; CV ≥ 0.5, corresponding to highly variable genes.

### Data acquisition and processing

After scanning, array images were assessed by eye to confirm scanner alignment and the absence of significant bubbles or scratches on the chip surface. 3’/5’ ratios for GAPDH and beta-actin were confirmed to be within acceptable limits (0.70–1.64), and BioB spike controls were found to be present on all chips, with BioC, BioD and CreX also present in increasing intensity. Array scanning data (CEL files) were processed using the RMA algorithm [48]. GeneSpring software (Silicon Genetics, Redwood City, CA) was used for data mining. Raw expression data were normalized per gene by dividing each measurement for each gene by the median of that gene’s measurements in the corresponding control non-trisomic samples. Normalized data were log-transformed. To reduce the noise and the variability induced by several sources including the manufacturing processes and the experimental procedures [49], expression data were pre-filtered and genes were considered suitable for differential evaluation if called present in at least 4 out of 6 samples. Microarray data were submitted to ArrayExpress (http://www.ebi.ac.uk/arrayexpress/) database (accession n. E-MTAB-1238). Statistical evaluation was performed by Welch t-test, corrected with Benjamini-Hochberg FDR algorithm and filtered for fold-changes in DS vs controls ≥ 1.2. Statistical significance was assessed at 0.05 and, for a further analysis, at 0.01. Supervised classification of samples was performed using hierarchical clustering (GeneSpring software).

### Bioinformatics data analyses

GO and pathway analyses of gene lists were performed using David Bioinformatics software (http://david.abcc.ncifcrf.gov/) [50]. For GO functional class scoring, a modified Fisher exact test (EASE score) was used and the statistical significance was set at p-value < 0.05. The fold enrichment (FE) value was used as a measure of the magnitude of enrichment. To compare results and to highlight more reliable GO classes, a second web-based software was used, the Gene Ontology Tree Machine (GOTM) [51]. To identify compounds with molecular signatures that might mitigate the effects of trisomy 21, Connectivity Map build 0.2 was used. The database contains 564 expression profiles representing the effects of 164 compounds on 4 cancer cell lines, using the Affymetrix U133 microarrays [52]. Because the U133 plus 2.0 array contains a greater number of probe-sets, the Connectivity Map analysis was performed using only the probe-sets common to both arrays. A list of perturbagens, hypothetically connected (positively and negatively) with the signature of interest was generated according to the permutation p. Perturbagenes with negative enrichment scores (< −0.7) were considered connected to the reversal or repression of the biological state encoded in the query signature. Perturbagens with enrichment scores < −0.7 and p-value < 0.05 were entered in the PASSonline software (Prediction of Activity Spectra for Substances) [53,54] in order to estimate their predicted activity. The current version of PASS available online (http://www.pharmaexpert.ru/passonline/index.php) predicts around 3750 pharmacological effects, biochemical mechanisms of action, specific toxicities and metabolic terms on the basis of structural formulae of drug-like substances with average accuracy ~ 95%. The predicted activity spectrum of a compound is estimated as probable activity (Pa) and probable inactivity (Pi) and only activities with Pa > Pi are considered. When Pa > 0.7, there is a high chance to confirm the activity experimentally, while if 0.5 < Pa < 0.7, the probability is lower but there is more than 50% chance for the compound to be a novel therapeutic molecule. Only compounds with Pa > 0.5 were considered here as potential therapeutic molecules.

### Quantitative Real-Time PCR

Single stranded cDNA was synthesized with random hexamer primers starting from 2 μg of total RNA using the High Capacity cDNA Archive Kit (Applied Biosystems). Real-time PCR was performed using 2 μl of cDNA and TaqMan Universal PCR MasterMix 2X on the Applied Biosystems 7300, according to the manufacturer’s procedures. PCR reactions were performed in triplicate. Beta-2-microglobulin and ring finger protein 111 (RNF111) housekeeping genes were chosen as reference genes [55,56].

### Protein extraction

Total protein extraction was carried out by cell incubation with the Tropix lysis solution (Applied Biosystems) supplemented with 0.1 M DTT in the presence of protease inhibitor cocktail 1X (Sigma). To better visualize protein-linked ubiquitins, lysis solution was supplemented with 5 mM manganese and 50 μM MG132 according to Mimnaugh and Neckers [57]. Cytoplasmic and nuclear fractions were obtained by celLytic NucLEAR Extraction kit (Sigma).

### Immunoprecipitation

Immunoprecipitation was carried out incubating 500 μg – 1 mg cytoplasmic extracts with specific antibodies at 4°C for 2 hours. Dynabeads Protein G (Invitrogen) were added to the mixture and the incubation was continued for other 2 hours. Proteins complexed with antibody - Dynabeads Protein G were immunoprecipitated and washed for three times with the use of a magnet, and finally eluted by boiling in SDS sample buffer for 10 minutes.

### Western blot analysis

Protein extracts (25–45 μg) were separated by 7-10% SDS-PAGE and then transferred on PVDF membrane (Millipore). Membranes were then blocked for 1 hour in 5% milk-1X PBS-0.1% Tween-20 (T-PBS) and then incubated from 4 hours to overnight with dilutions of specific primary antibodies: 1:1000 anti-NFATc1 (H-110), 1:100 anti-NFATc2 (4G6-G5), 1:1000 anti-NFATc3 (M-75), 1:1000 anti-NFATc4 (H-74), 1:2500 anti-DYRK1A (H-143) (SantaCruz); 1:1000 anti-DSCR1 (N-20) (SIGMA); 1:2000 anti-NF-*k*B p65 (Upstate); 1:1000 anti-NF-*k*B p50 (Upstate); 1:1500 anti-I*k*B-alfa (L35A5), 1:1000 anti-pI*k*B-alfa (Ser32/36), 1:1000 anti-ubiquitin (P4D1) (CellSignaling). 1:2000 anti-α-Tubulin (SIGMA) and 1:1000 Histone H1 (SPM 256) (SantaCruz) were used to normalize the levels of total, cytosolic and nuclear proteins. As secondary antibodies, 1:5000 anti-mouse, 1:2000 anti-rabbit and 1:2000 anti-goat IgG horseradish peroxidase conjugated (GE-Healthcare) were used and the specific bands were visualized by ECL plus reaction (GE-Healthcare).

### Luciferase assay

To test NF-*k*B transcriptional activity, LCLs from DS subjects and controls were plated in 24-well plates at density of 5x10^5^ cells/well and transfected, 24 hours later, with pNF-*k*B-Luc (Path Detect NF-*k*B Cis-Reporting System, Stratagene) and pSV-β-Gal plasmid by using Turbofect Transfection Reagent (Fermentas), according to the manufacturer’s instruction. Forty-eight hours post-transfection, cells were harvested and processed to evaluate the luciferase and β-galactosidase activities by using Dual Light Luciferase System (Tropix, Bedford, MA, USA) in a bioluminometer (TECAN, Infinite 200). Experiments were performed in triplicate and repeated independently twice. The ratio of firefly luciferase activity to β-galactosidase activity was expressed as relative light units (RLU).

### Luminescent proteasome assay

To individually measure the chymotrypsin-, trypsin- and caspase-like proteasome activities in cultured cells, the Proteasome-Glo™ 3 substrates Cell-Based Assay (Promega) was used. LCLs were plated in 96-well plates at concentration of 2x10^4^/well and incubated at 37°C overnight. The day after, cells were treated with 100 μM MG132 for 2 hours (inhibitor control) and 0.1 mM H_2_O_2_ for 30 minutes (test treatment) before adding the three specific proteasome substrates: Such-LLVY-Glow™ Substrate for the Chymotrypsin-Like activity, Z-LRR-Glow™ Substrate for the Trypsin-Like activity and Z-nLPnLD-Glo™ Substrate for the Caspase-Like activity. Luminescence for each sample, performed in duplicate and in two separate experiments, was read after 15 minutes in a plate-reading luminometer (TECAN, Infinite 200).

### Statistical analysis

Data were reported as means ± S.E. Statistical analysis was performed with SPSS 13.0 software and the student’s t-test was used for the statistical significance (two-tailed, p < 0.05) of differences between means of DS and control subjects. To validate the microarray data, the average expression ratios (DS/controls) of both array and qRT-PCR were subjected to Pearson’s correlation analysis.

## Results

### Supervised analysis of pooled data of DS and control samples

For the analysis of differentially expressed genes, six DS LCLs were compared to six control samples. A first list of 3,416 differentially expressed genes was generated by using a corrected p-value cut-off of 0.05. Of these, 1,051 (30.7%) were up-regulated and 2,368 (69.3%) were down-regulated, with a gene expression variation ranging from 0.67 to 3.04. No chr 21 gene resulted down-regulated. A second list of genes was then generated by using a corrected p-value cut-off of 0.01. In this case, a total of 406 transcripts resulted significantly either up-regulated (n = 71; 17.5%) or down-regulated (n = 335; 82.5%) (Additional file 1A). Supervised hierarchical clustering of both the 4,490 and the 406 transcripts clearly distinguished between DS and control samples (Additional file 1B, C).

### Chr 21 expression profiles

**Figure 1:**
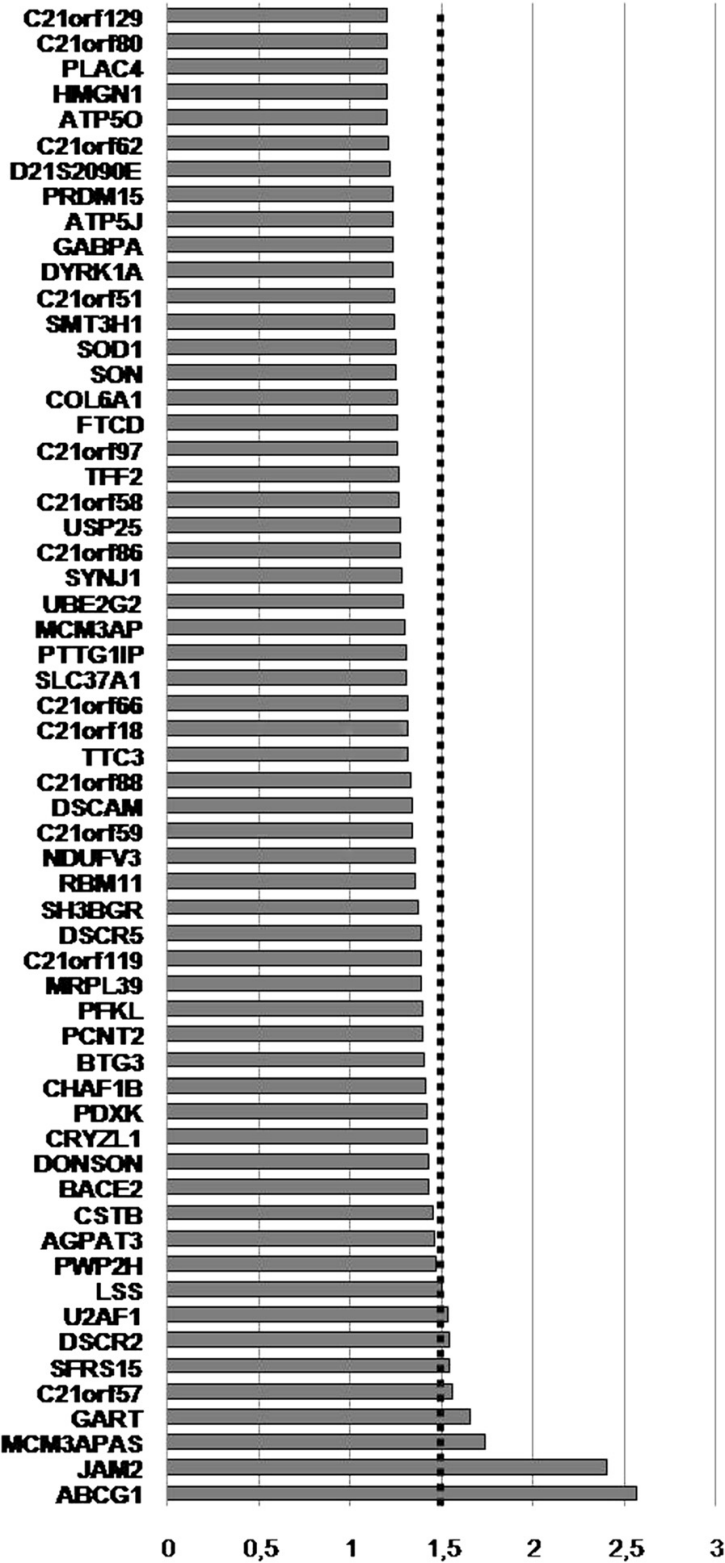
**Average up-regulation ratios of chromosome 21 differentially expressed genes (*p < 0.05*).**

Two hundred-twenty genes out of 449 genes annotated on chr 21 (NCBI RefSeq 37.2) were detected in this microarray analysis. Among these, 59 known genes (13.1% of total chr 21 annotated genes) resulted differentially expressed in DS vs controls (p < 0.05, FC ≥ 1.2), with FC ranging from 1.20 to 2.57 and a mean DS/control ratio of 1.36 ± 0.2. A graphic view of the differentially expressed chr 21 genes is illustrated in Figure 1, that shows how a substantial number of triplicated genes escapes the gene-dosage rule. To identify functional categories associated with the trisomic genes, the list of chr 21 deregulated genes was submitted to a GO analysis. Results indicated ‘*ATPase activity coupled to transmembrane movement of substances*’ (p = 0.005, FE 11.3), ‘*cofactor metabolic proces*s’ (p = 0.029, FE 5.8), ‘*regulation of cholesterol biosynthetic process*’ (p = 0.031, FE 62.2) and ‘*oxidative phosphorylation’* (p = 0.04, FE 9.2) as the major enriched categories (Table 1). The analysis of the same genes, performed using KEGG database, showed Parkinson’s (p = 0.01, FE 8.3), Alzheimer’s (p = 0.02, FE 6.1) and Huntington’s (p = 0.03, FE 5.5) diseases as the most deregulated biological pathways (Table 2).

**Table 1:** Enriched GO categories of chromosome 21 up-regulated genes

** GO term**	**Genes**	%*	**p-value**	**FE**
ATPase activity, coupled to transmembrane movement of substances	ATP5O	6.2	0.005	11.3
	ATP5J			
	ABCG1			
Cofactor metabolic process	SOD1	6.2	0.029	5.8
	FTCD			
	PDXK			
	CRYZL1			
Regulation of cholesterol biosynthetic process	ABCG1	3.1	0.031	62.2
	SOD1			
Oxidative phosphorylation	ATP5O	4.7	0.04	9.2
	ATP5J			
	NDUFV3			

*FE* fold enrichment, *% of genes in category.

**Table 2:** Enriched pathways of chromosome 21 up-regulated genes

** Pathway term**	**Genes**	**%**	**p-value**	**FE**
Parkinson’s disease	ATP5O	6.2	0.010	8.3
	ATP5J			
	NDUFV3			
	UBC6/7			
Alzheimer’s disease	ATP5O	6.2	0.023	6.1
	ATP5J			
	NDUFV3			
	BACE			
Huntington’s disease	ATP5O	6.2	0.030	5.5
	ATP5J			
	NDUFV3			
	SOD1			

*FE* fold enrichment.

### Analysis of chr 21 gene expression variability among DS and controls

To assess the expression variation of chr 21 genes among DS and control samples, we focused the analysis on the variability of all chr 21 transcripts present on the array with FC ≥ 1.2 (n = 167). Among controls, 77 transcripts (46.1%) had CV ≤ 0.2, 74 (44.3%) had 0.2 < CV < 0.5 and 16 (9,6%) had CV ≥ 0.5. Among DS samples, 110 transcripts (65.9%) had CV ≤ 0.2, 48 (28.7%) had 0.2 < CV < 0.5 and 9 (5.4%) had CV ≥ 0.5 (Table 3). Fifty-five transcripts were tightly regulated in both DS and controls and among them, notably, mitochondrial-related genes (ATP5O, ATP5J and NDUFV3), SOD1, DYRK1A and cell-cycle related genes (Additional file 2). GO analysis of this gene subset revealed the enrichment of the following categories: ‘*Cofactor metabolic process’* (p = 0.007, FE 9.4), *‘ATPase activity, coupled to movement of substances’* (p = 0.0018; FE 15.9), *‘oxidative phosphorylation*’ (p = 0.016, FE 14.9), *‘mitochondrial proton-transporting ATP synthase complex, coupling factor F(o)’* (p = 0.016, FE 120.4), *‘regulation of cholesterol biosynthetic process’* (p = 0.019, FE 100.3) (Table 4). KEGG database showed the enrichment of ‘*oxidative phosphorylation’* (p = 0.03, FE 9.9) besides Parkinson’s, Huntington’s and Alzheimer’s diseases (Table 5). Neither GO enriched categories nor KEGG pathways were associated to the 22 transcripts tightly regulated in controls (CV ≤ 0.2) and with little variation in DS (0.2 < CV < 0.5).

**Table 3:** Chromosome 21 transcripts sorted by the coefficient of variation in both DS and control samples

			**DS samples**		
		**CV ≤ 0.2**	** 0.2 < CV < 0.5**	**CV ≥ 0.5**	**TOTAL**
	CV ≤ 0.2	55	22	0	77
**Controls**	0.2 < CV < 0.5	49	19	6	74
	CV ≥ 0.5	6	7	3	16
	TOTAL	110	48	9	167

*CV* coefficient of variation, *DS* Down syndrome.

**Table 4:** GO categories of the 55 chromosome 21 transcripts tightly regulated in DS and controls (CV ≤ 0.2)

** GO term**	**Genes**	%*	**p-value**	**FE**
Cofactor metabolic process	SOD1	9.5	0.0077	9.4
	FTCD			
	PDXK			
	CRYZL1			
ATPase activity, coupled to transmembrane movement of substances	ATP5O	9.5	0.0018	15.9
	ATP5J			
	ABCG1			
Oxidative phosphorylation	ATP5O	7.1	0.016	14.9
	ATP5J			
	NDUFV3			
Mitochondrial proton-transporting ATP synthase complex, coupling factor F(o)	ATP5O	4.8	0.016	120.4
	ATP5J			
Regulation of cholesterol biosynthetic process	ABCG1	4.8	0.019	100.3
	SOD1			

*FE* fold enrichment, *% of genes in category.

**Table 5:** Enriched pathways of the 55 chromosome 21 transcripts tightly regulated in DS and controls (CV ≤ 0.2)

** Pathway term**	** Genes**	** %**	**p-value**	** FE**
Parkinson’s disease	ATP5O	9.5	0.002	13.8
	ATP5J			
	NDUFV3			
	UBC6/7			
Huntington’s disease	ATP5O	9.5	0.0062	9.2
	ATP5J			
	NDUFV3			
	SOD1			
Oxidative phosphorylation	ATP5O	7.1	0.03	9.9
	ATP5J			
	NDUFV3			
Alzheimer’s disease	ATP5O	7.1	0.049	7.6
	ATP5J			
	NDUFV3			

FE, fold enrichment.

### Genome-wide expression analysis, functional classes and pathway perturbation

GO functional class scoring was performed by comparing the list of the 406 differentially expressed genes to the complete list of genes spotted on the array.

Our analysis revealed a down-regulation of the biological processes related to ubiquitin metabolism; cell signalling, with a particular enrichment for the NF-*k*B cascade; cell cycle; protein localization; regulation of gene expression (Table 6). Among up-regulated categories, the most disrupted were those related to developmental processes and to transport and localization, with a strong enrichment of calcium ion transport (Table 7).

**Table 6:** Enriched GO categories of down-regulated genes (p < 0.01), sorted by p-value

** Biological process**	**GO term**	**N. of genes**	%*	**p-value**	**FE**
*Ubiquitin metabolism*	ubiquitin-dependent protein catabolic process	16	5.4	5.2E-6	4.3
	modification-dependent protein catabolic process	25	8.5	1.4E-5	2.7
	proteolysis involved in cellular protein catabolic process	25	8.5	2.9E-5	2.6
	ligase activity	17	5.8	0.001	2.6
	proteolysis	29	9.8	0.009	1.6
	ubiquitin-protein ligase activity	8	2.7	0.013	3.2
	proteasomal ubiquitin-dependent protein catabolic process	6	2.0	0.025	3.6
*Cell signalling*	positive regulation of I-*k*B kinase/NF-*k*B cascade	9	3.1	0.0002	5.5
	positive regulation of protein kinase cascade	10	3.4	0.0024	3.5
	positive regulation of signal transduction	12	4.1	0.01	2.4
*Protein localization*	establishment of protein localization	25	8.5	0.002	2.0
*Cell cycle*	mitotic cell cycle	14	4.7	0.008	2.3
	cell cycle phase	15	5.1	0.009	2.2
	M phase of mitotic cell cycle	10	3.4	0.013	2.7
	mitosis	9	3.1	0.031	2.4
	nuclear division	9	3.1	0.031	2.4
	interphase	6	2.0	0.033	3.3
*Regulation of gene expression*	gene expression	62	21.0	0.022	1.3
	mRNA processing	12	4.1	0.023	2.2
	RNA splicing	11	3.7	0.024	2.3
*Vescicle related*	ER to Golgi vesicle-mediated transport	4	1.4	0.033	5.6

*FE* fold enrichment, *% of genes in category.

**Table 7:** Enriched GO categories of the up-regulated genes (p < 0.01), sorted by p-value

**Biological process**	** GO term**	**N. of genes**	%*	**p-value**	**FE**
*Transport and localization*	calcium ion transport	5	8.3	0.001	10.6
	transmembrane transport	7	11.7	0.01	3.7
	metal ion transport	6	10.0	0.016	3.9
	localization	17	28.3	0.022	1.7
	cation transport	6	10.0	0.032	3.3
	transport	15	25.0	0.034	1.7
	ion transport	7	11.7	0.035	2.8
	establishment of localization	15	25.0	0.037	1.7
*Development*	cellular developmental process	14	23.3	0.002	2.5
	anatomical structure development	17	28.3	0.004	2.0
	cell differentiation	13	21.7	0.005	2.4
	system development	16	26.7	0.005	2.1
	multicellular organismal development	17	28.3	0.015	1.8
	organ development	12	20.0	0.021	2.1

*FE* fold enrichment, *% of genes in category.

Pathway analysis identified ubiquitin-mediated proteolysis as the pathway mostly influenced by trisomy 21 (p = 2.9E-5; FE 4.8). Among genes involved in the ubiquitin-dependent proteolysis, a number of E2-conjugating enzymes (UBE2A, UBE2B, UBE2H), E3-ligases (UBE3A, ITCH, SMURF2, F-box proteins, MIB1) and deubiquitinating enzymes (USP1, USP2, USP8, USP12, USP15, USP28, USP32, USP33, USP34, USP38, USP47) resulted down-regulated. Microarray data were validated by qRT-PCR on 11 differentially expressed genes with different functions and by comparing their average expression ratios (DS/controls) with those of the array (Additional file 3).

### Selection of possible therapeutic compounds

Connectivity map and PASS-assisted exploration were used to identify compounds whose molecular signatures counteracted those of DS lymphoblasts and to predict their therapeutic potential, respectively. We found 17 compounds with enrichment score < −0.7 (Additional file 4). Among these, adiphenine, eticlopride and vigabatrin display a predicted proteasome ATPase inhibitor activity (Pa score 0.72, 0.65 and 0.59, respectively). Adiphenine also displays a putative ubiquitin thiolesterase inhibitor and a proteasome endopeptidase complex inhibitor activity (Pa score 0.58 and 0.56, respectively). Finally, oxybenzone display a free radical scavenger activity (Pa 0.79).

### Validation of some significantly deregulated GO categories

#### Protein ubiquitination and proteasome activity

**Figure 2:**
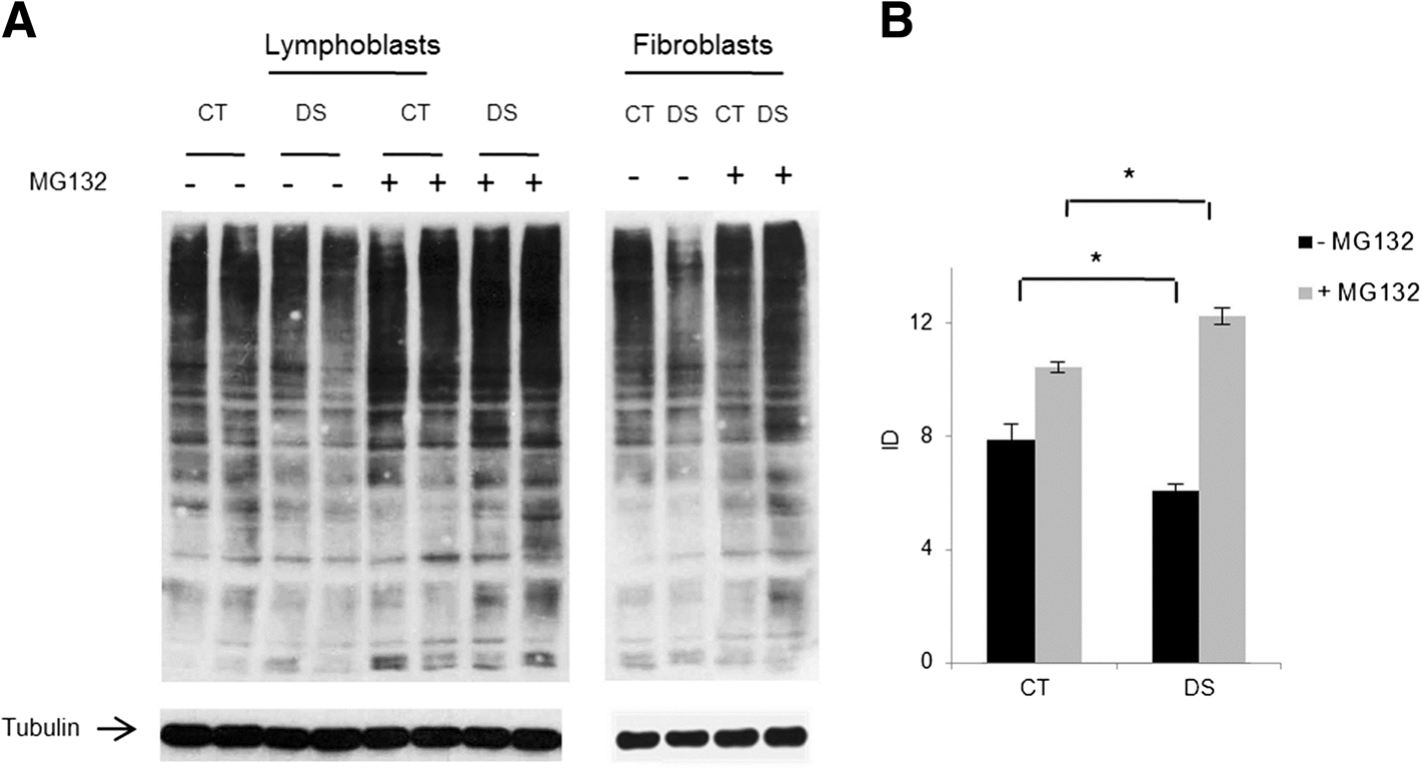
**Western blot analysis of total ubiquitin-bound protein levels. A**) Western blot performed on lymphoblasts and fetal fibroblasts in basal conditions and following proteasome blocking (40 μM MG132 for six hours). **B**) Histograms show the average protein ubiquitination of lymphoblasts derived from two controls and two DS subjects, normalized on tubulin levels. * *p < 0.05*.

Ubiquitinated protein levels were measured by Western blot with or without proteasome block by MG132. Results in both LCLs and fetal fibroblasts showed a reduction of the ubiquitination state in DS resting cells and an increase following MG132 treatment (Figure 2A, B).

**Figure 3:**
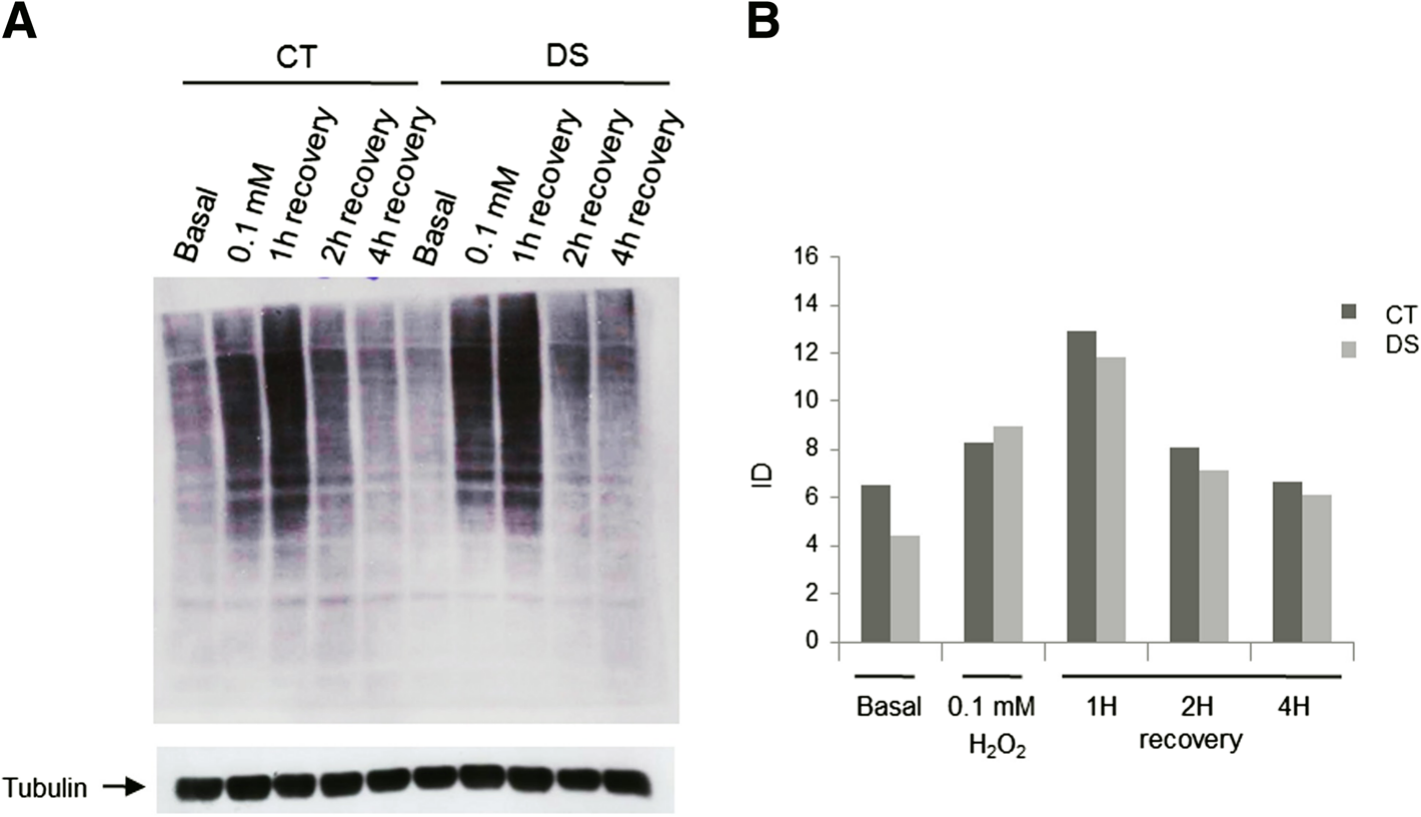
**Ubiquitin-bound proteins in LCLs from a DS and a control subject in basal and OS conditions. A**) Western blot analysis of ubiquitin-bound proteins in lymphoblasts from a DS and a control subject in basal conditions, under oxidative stress (0,1 mM H_2_O_2_ for 30 minutes) and after 1, 2 and 4 hours of recovery. **B**) relative densitometry.

**Figure 4:**
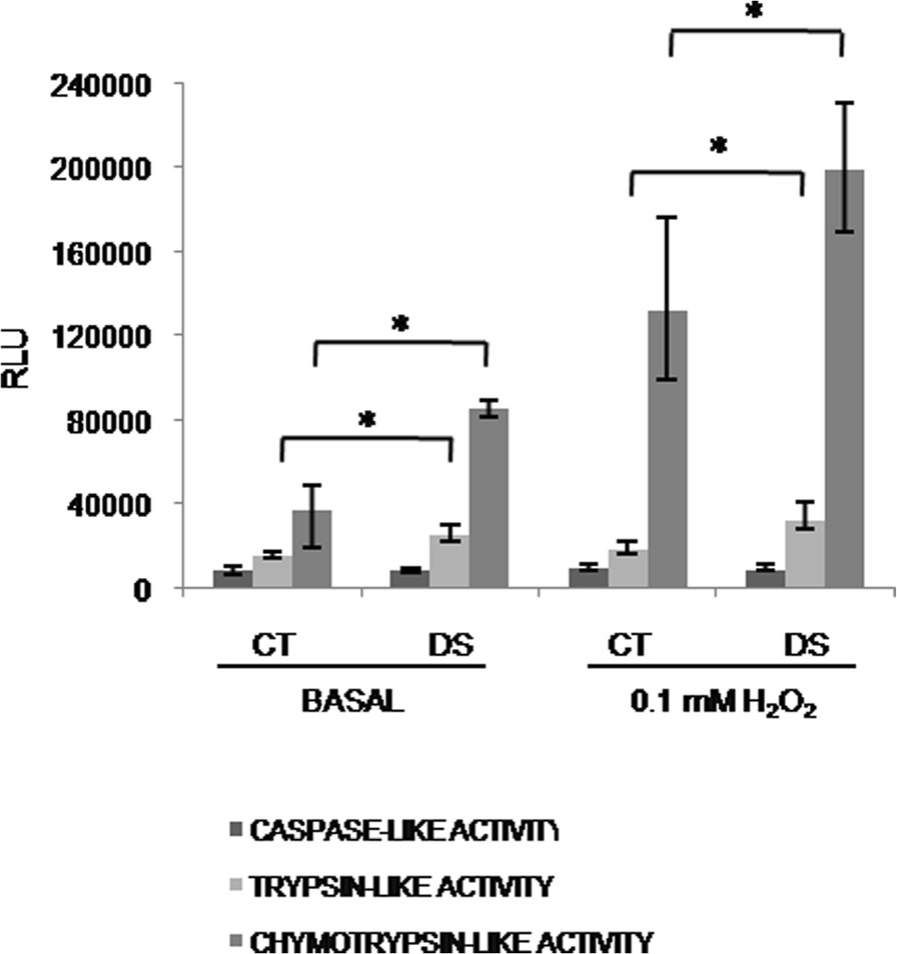
**Proteasome activity assay from LCLs, in basal conditions and after treatment with 0,1 mM H**_**2**_**O**_**2 **_**for 30 minutes.** Histograms show the average of the activities of DS subjects (n = 3) versus controls (n = 3), evaluated in duplicate and repeated twice. * *p < 0.05*.

As chronic oxidative stress (OS) is a feature of DS and a defective response to OS is known to occur in DS with accumulation of protein damage [58], ubiquitinated protein levels were also measured after incubation with 0.1 mM H_2_O_2._ DS cells showed a trend to increase ubiquitin-bound proteins after OS induction. After H_2_O_2_ withdrawal, DS cells showed a more efficient recovery in the first two hours compared to controls (Figure 3). As the increase of ubiquitination following proteasome blocking suggested a possible involvement of the proteasome, the three proteasome activities were tested in LCLs before and after incubation with H_2_O_2_. Proteasome activity assay revealed a significant increase in the trypsin-like and in the chymotrypsin-like activities (p < 0.01) in DS subjects, both in basal and OS conditions, while no differences were observed for the caspase-like activity (Figure 4).

#### IkB-alfa/NF-kB

**Figure 5:**
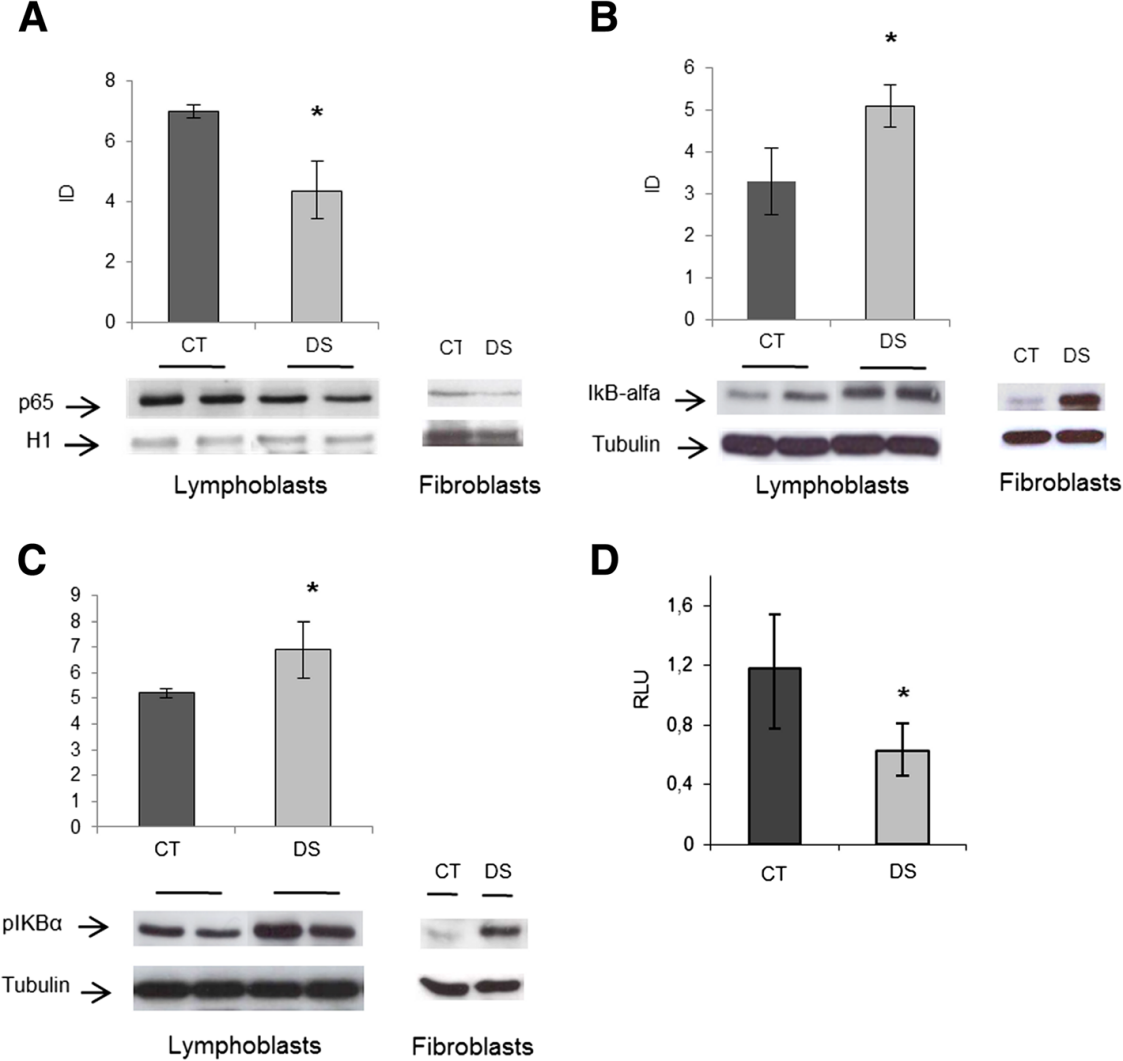
**NF-kB p65, IkB-alfa and phospho-IkB-alfa in LCLs and fibroblats and relative densitometry. A**) Western blot of NF-*k*B p65 nuclear levels and **B-C**) I*k*B-alfa and phospho-I*k*B-alfa cytosolic levels LCLs and relative densitometry. p65, I*k*B-alfa and phospho-I*k*B-alfa protein levels in fetal fibroblasts from one DS subject and one control are shown as well. As internal controls, tubulin and H1 histone were used for cytosolic and nuclear marker proteins, respectively. **D**) NF-*k*B Luciferase assay in lymphoblasts. Histograms show the average of the activity, expressed as relative light units (RLU), of DS subjects (n = 3) versus controls (n = 3) normalized on the average of the b-galactosidase activity. Experiments were performed in triplicate and repeated twice. * *p < 0.05*.

**Figure 6:**
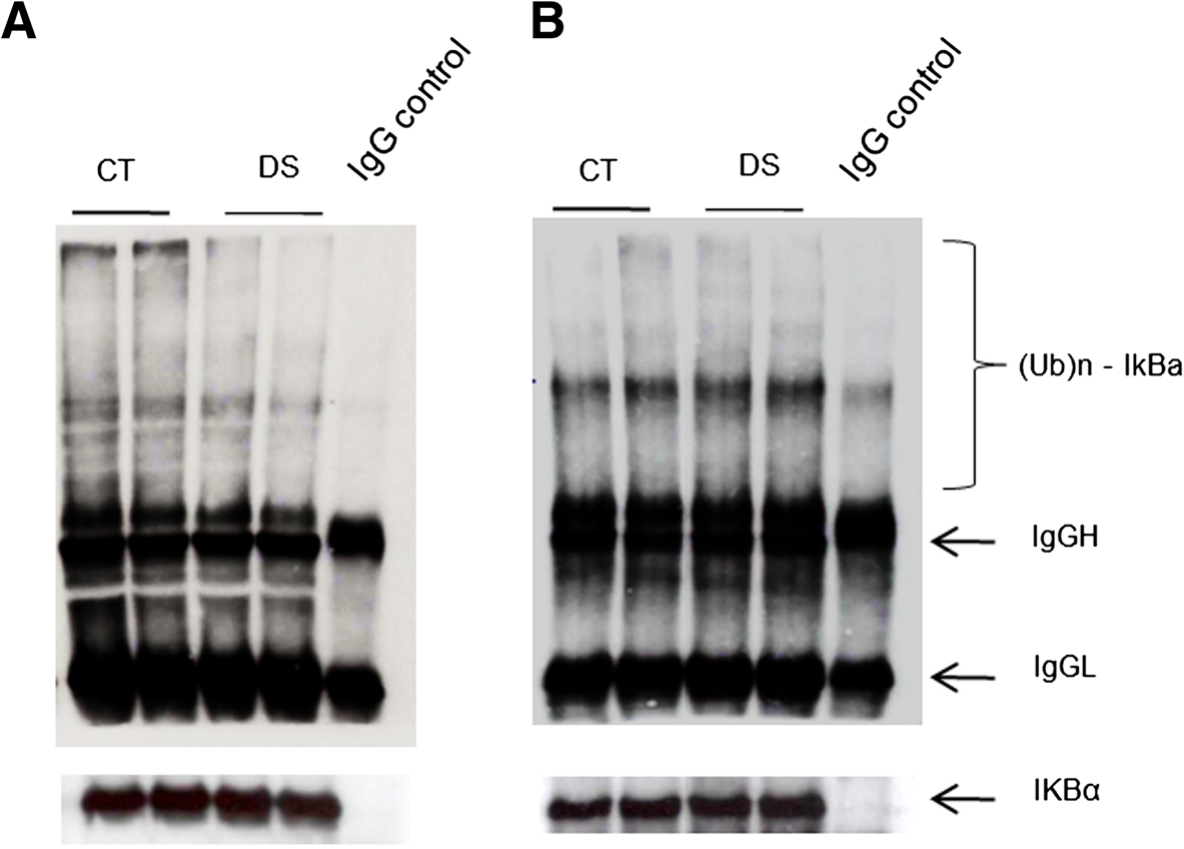
**Ubiquitination of I*k*B-alfa in basal conditions** (**A**) **and after proteasome blocking with 40 μM MG132 for six hours** (**B**). Cytoplasmic extracts were immunoprecipitated for I*k*B-alfa and then analyzed by western blot using an antibody against ubiquitin. IgG H and IgG L indicate immunoglobulin heavy and light chains in the immunoprecipitates that cross-react with the secondary antibody.

NF-*k*B is finely regulated by a complex network of gene products and post-translational modifications. To demonstrate if a NF-*k*B defect occurs in DS, we choose to analyse the down-stream product of the NF-*k*B cascade, i.e. the behaviour of p50/p65 and of its inhibitors in DS cell lines. Levels of p50, p65, I*k*B-alfa and phospho-I*k*B-alfa proteins were assessed by Western blot using specific antibodies in DS and control samples. Results showed a significant reduction of p65 levels in the nuclear fractions and a significant increase of I*k*B-alfa in the cytosolic fractions of DS subjects, in both the dephosphorylated and phosphorylated forms (Figure 5A, B, C). No difference was observed for NF-*k*B p50 subunit levels (data not shown). Luciferase assay confirmed a significant reduction of NF-*k*B transcriptional activity in DS LCLs (p < 0.05) (Figure 5D). Immunoprecipitation of I*k*B-alfa in cytosolic fractions and subsequent Western blot with specific anti-ubiquitin antibody showed a reduction of the ubiquitinated levels of this inhibitor under basal conditions in DS fractions (Figure 6A). Ubiquitination levels returned similar to controls after proteasome blocking (Figure 6B).

#### DYRK1A/DSCR1/NFAT genes

**Figure 7:**
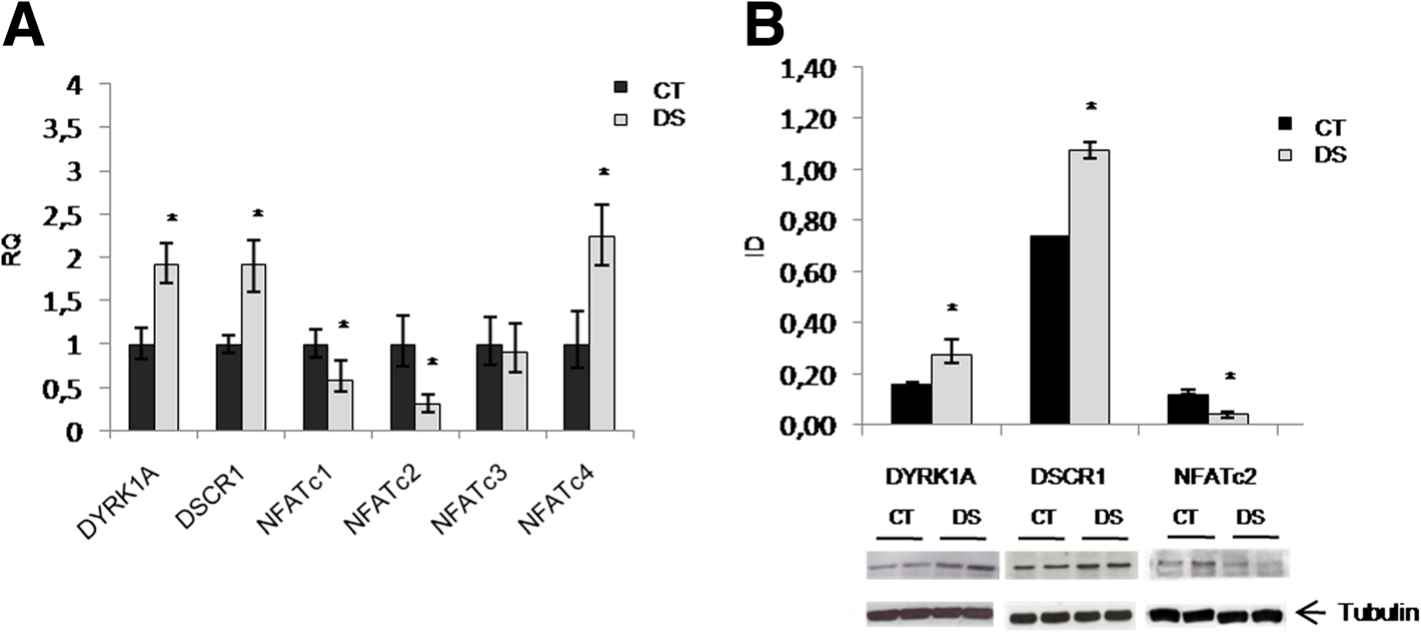
**Analysis of DYRK1A, DSCR1 and NFATc1-4 gene products in LCLs. A**) Relative quantification. Histograms show mRNA levels of pools of DS subjects (n = 3) versus controls (n = 3). All data are expressed relatively to control subjects, to which is assigned a value of 1-fold (1x). **B**) Western blot of total protein levels. * *p < 0.05*.

**Figure 8:**
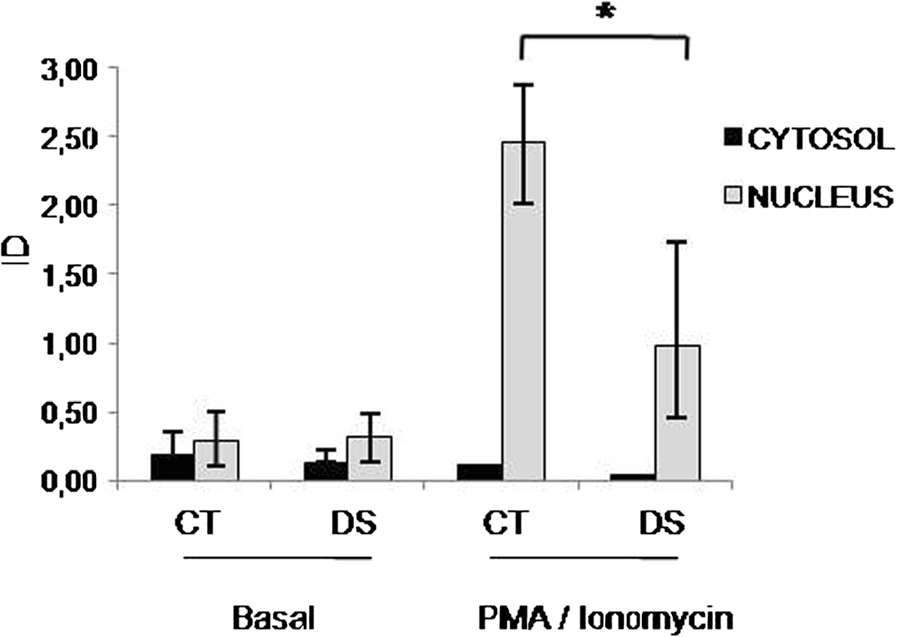
**Analysis of activation and nuclear translocation of NFATc2, induced by PMA/Ionomycin.** As internal controls, tubulin and H1 histone were used for cytosolic and nuclear marker proteins, respectively. * *p < 0.05*.

DYRK1A, DSCR1 and the members of the NFAT transcription factors were evaluated by RT-PCR and Western blot. We observed a significant upregulation of DYRK1A, DSCR1 and NFATc4 (about 2-fold increase) and a down-regulation of NFATc2 (69% reduction) and NFATc1 (49% reduction) expression profiles in DS LCLs compared to controls (Figure 7A). No difference was observed for NFATc3. At protein level, we confirmed the significant reduction of NFATc2 and the increase of DYRK1A and DSCR1 (Figure 7B) while no difference was observed for NFATc1 and NFATc4 (data not shown). To assess NFAT nuclear translocation, Western blot experiments were performed upon induction of calcium fluxes through PMA/ionomycin. Results showed a reduction of NFATc2 nuclear levels following stimulation, suggesting the presence of mechanisms acting to inhibit its translocation (Figure 8).

**Figure 9:**
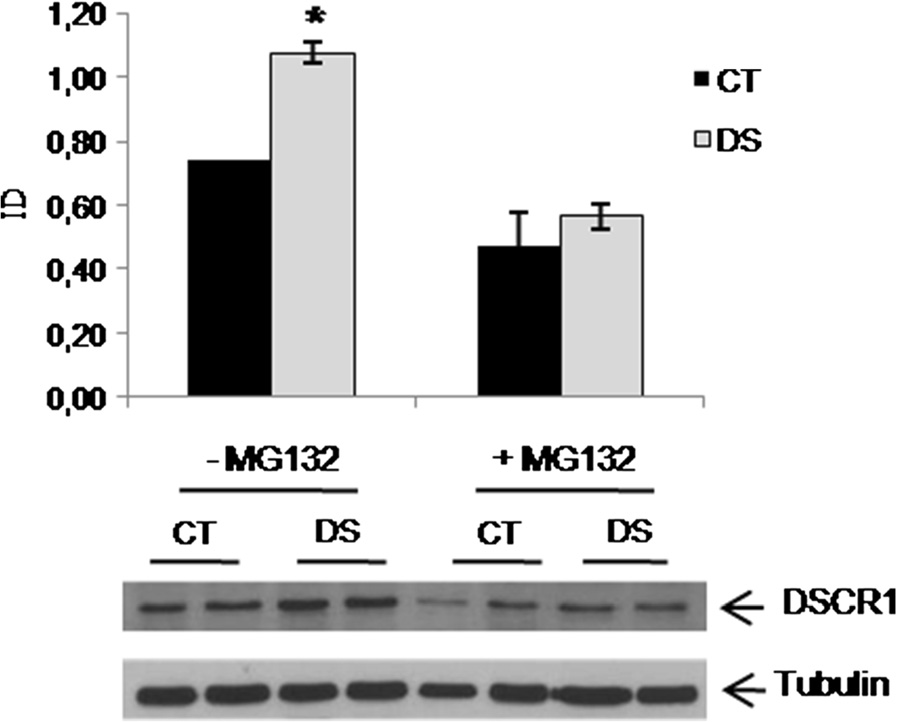
Western blot analysis of DSCR1 protein levels in basal conditions and following proteasome blocking with 40 μM MG132 for six hours in lymphoblasts from 2 DS and 2 control subjects. * *p < 0.05.*

Finally, soluble DSCR1 protein levels were investigated before and after proteasome blocking. Results showed a reduction of soluble DSCR1 levels in both samples after MG132 treatment, with a more marked effect in DS subjects where the DSCR1 levels became comparable to controls (Figure 9).

## Discussion

In spite of the great efforts made to analyze the effects of dosage imbalance at molecular level, the pathogenesis of DS is still unclear. So far, expression studies performed with distinct approaches led to inconclusive results, thus underlining the importance of integrating microarray data with functional validation of selected pathways.

The present study was aimed at analyzing gene expression profiles in LCLs from DS and controls to identify genes and metabolic pathways involved in DS pathogenesis, with an emphasis toward chr 21 genes. To select reliable GO classes, two distinct web-based softwares working on different statistic algorithms (DavidBioinformatics and GOTM) were used. These GO classes where further analysed to provide experimental evidence of their disruption in DS.

Despite some inherent limitations, the utility of LCLs is increasingly recognized in both genetic and functional studies [59]. Considering the similarity of the expression and regulation of certain genes between LCLs and neurons, LCLs have been used as surrogate cells in studies of neurological disorders as in the case of Parkinson’s disease [60,61] and Alzheimer’s disease [62]. LCLs have also been successfully used to study mitochondrial and organellar dysfunction as in the case of autophagy in juvenile neuronal ceroid lipofuscinosis [63]. Beside the present study, LCLs have been used in expression studies of chr 21 genes in three previous works [21,23,38] where it was demonstrated that the variance on gene expression levels due to culture conditions is extremely low in this cell model [21]. Moreover, Merla et al., [64] established six independent LCLs for the same individual and compared expression levels for 25 chr 21 genes finding a strong correlation ranging from 0.8 to 0.92. In this study, to maximize the quality of LCLs, each aliquot of LCLs was maintained in culture for no longer than 3 months (less than 160 ‘population doubling levels’) and cells were karyotyped before microarray analysis and before each experimental validation to exclude the possibility of chromosomal rearrangements (other than full trisomy 21).

### Overexpression of chr 21 genes

Our results showed a significant up-regulation of a subset of 59 chr 21 genes (p < 0.05) with a mean DS/control ratio of 1.36 ± 0.2. This confirms that most trisomic genes escape the predicted gene-dosage rule of 1.5-fold increase and underscores the existence of complex and unravelled mechanisms of transcriptional regulation [65]. In the present study, GO analysis of the overexpressed chr 21 genes indicated ATPase activity coupled to transmembrane movement of substances and oxidative phosphorylation as the major enriched categories. This result is in agreement with previous gene expression studies in fetal DS cerebral cortex, heart and trophoblasts [5,7,13,15] in which the most consistently disregulated chr 21 genes were those involved in mitochondrial function related metabolic pathways, such as ATP5O and ATP5J (encoding two mitochondrial ATP synthase subunits), NDUFV3 (subunit of NADH dehydrogenase), and SOD1 (involved in antioxidant defense system). Mitochondrial dysfunction has been proposed in the pathogenesis of DS for years. In particular, deficit of oxidative phosphorylation due to a deregulation of the mitochondrial respiratory chain complexes I, III and V [34,35,66-68], increased levels of the Krebs cycle enzymes (aconitase and NADP-linked isocitrate dehydrogenase) [69] and impaired mtDNA repair systems [70] were described in human DS cells. In isolated mitochondria from DS mice cortex, a decreased membrane potential and ATP content and selective defects in Complex-I mediated respiration were also found [71]. Furthermore, a close relationship between mitochondrial abnormalities and oxidative damage has been described in DS brains and fibroblasts [72]. Mitochondrial dysfunctions can result either in decreased ATP levels, or in increased ROS production [35,73]. Mitochondrial dysfunction was also described in neurodegenerative diseases such as Alzheimer’s, Parkinson’s and Huntington’s diseases and in the normal aging processes [73-77]. Interestingly, when the trisomic genes, upregulated in the present gene expression study, were submitted to pathway analysis, Alzheimer’s, Parkinson’s and Huntington’s diseases were identified as the most deregulated, in agreement with the meta-analysis from heterogeneous human and mouse DS data sets [27]. Finally, when only the amplified chr 21 genes tightly regulated in DS and in control subjects were submitted to *in silico* analysis, the same categories and pathways were identified. Taken together, these findings suggest that genes directly or indirectly involved in the oxidative phosphorylation, ATPase activity and, in general, in mitochondrial function, may have a role in the pathogenesis of DS phenotypes.

### Genome-wide deregulation and pathway perturbation induced by trisomy 21

The genome-wide expression analysis revealed that 30.7% and 17.5% of genes were up-regulated at p < 0.05 and p < 0.01, respectively. A low percentage of up-regulated genes was also reported in two previous studies on LCLs, showing that 29% and 39% of genes were up-regulated, respectively [21,23]. In this study, genes involved in ubiquitin metabolism, cell signalling (with a particular enrichment of NF-*k*B cascade), cell cycle, protein localization and regulation of gene expression were down-regulated, and genes involved in developmental processes and calcium ion transport were up-regulated. The latter results are in agreement with previous genome-wide studies that reported the impairment of GO categories associated to developmental processes, both in fetal and adult DS biomaterials [9,14], as well as of calcium/potassium signalling on cell-free mRNA from amniotic fluid [78].

At mRNA level, while the ubiquitin pathway was reported as deregulated in DS trophoblasts and in rRNA-depleted samples from DS endothelial progenitor cells (EPCs) [15,79], NF-*k*B cascade, linked to the ubiquitn-mediated proteolysis, was found down-regulated in the present study for the first time. The close relation between the ubiquitin-mediated proteolysis and NF-*k*B and their relevance to a wealth of human disorders make these two pathways attractive candidates for DS pathogenesis.

### The ubiquitin-dependent proteolysis

The UPS is the major proteolytic pathway used by eukaryotic cells to metabolize proteins [80]. Proteins to be degraded are covalently linked to a polyubiquitin chain by three different enzymes and then targeted to the 26S proteasome where are disassembled in small peptides, amino acids and ubiquitin monomers. UPS is known to metabolize misfolded, oxidized and damaged proteins, but also proteins involved in signal transduction, cell cycle regulation, differentiation and development, cellular response to stress, regulation of the immune and inflammatory response. A link between protein synthesis and degradation by UPS has been proposed [81]. Hence, derangements in this system, that can be associated to loss of function (mutation in an ubiquitin system enzyme or target substrate) or gain of function (abnormal or accelerated degradation of the target protein) could underlie, directly or indirectly, the pathogenesis of many diseases, including some features belonging to the DS spectrum. In particular, accumulation of ubiquitin conjugates and/or inclusion bodies associated with ubiquitins have been reported in a broad array of chronic neurodegenerative diseases, such as the neurofibrillary tangles of Alzheimer’s disease (AD), brain stem Lewy bodies (LBs) in Parkinson’s disease (PD) and intracellular bodies in Huntington’s disease [82]. In these cases, an involvement of the UPS was suggested [83-85]. So far, no data were available on the ubiquitination status and proteasome activity in human DS. By means of a protein screen by SDS-page, a study showed an increase of the proteasome zeta chain, an alpha subunit of the 20S proteasome, and of isopeptidase T, a deubiquitinating enzyme, in fetal DS brain [86]. More recently, a study on the cerebellum of Ts65Dn mice showed a reduction of the proteasome chymotrypsin-like activity and an increase of ubiquitinated proteins [87], results possibly ascribable to the increased levels of beta-amyloid found in Ts65Dn mice brains [88].

The present study in human cell lines shows for the first time that, under basal conditions, there is a reduction of ubiquitinated protein levels in DS. This observation may either depend on defective ubiquitin-protein ligase activity (as suggested by microarray data) or on increased proteasome function, as demonstrated by the increase of two out of the three proteasome activities. Under OS, we observed an increase of ubiquitinated proteins in DS compared to the control sample. In the recovery period, ubiquitinated proteins were efficiently cleared in DS, probably due to the observed increase of the proteasome activities during OS. Previous studies have shown that chronic OS is a feature of DS [35,36,71,89-92]. Increased protein damage has also been shown in DS [58]. These observations may explain the increased proteasome activity, the accumulation of Ub-bound proteins after proteasome blocking with MG132 and, at least in part, the reduction of ubiquitin-bound proteins. These findings offer a new perspective to study this system in association to relevant DS pathological features, such as neurodegeneration, autoimmune disorders and predisposition to cancer.

### I*k*B-alfa/NF-*k*B

The UPS is implicated in numerous cellular processes including activation of transcription factors such as NF-*k*B. The NF-*k*B family of transcription factors is critical in the development and maintenance of the immune system. Five NF-*k*B subunits exist and the most abundant and active form is a heterodimer composed of p50 and p65. Generally, this heterodimer is sequestered in the cytosol by one of the I*k*B inhibitors, most commonly I*k*B-alfa and is activated only upon I*k*B phosphorylation and subsequent proteasome mediated degradation [47]. Since this transcription factor is connected to a wide array of immune and inflammatory disorders, along with cell apoptosis and delay of cell growth, disruption of its pathway could play an important role in DS pathogenesis. In the present study, the GO analysis showed a down-regulation of the NF-*k*B cascade. Our experimental data provide the first evidence of a reduction of the NF-*k*B p65 subunit in DS nuclear fractions and of a significant reduction of its transcriptional activity. Along with this observation, cytosolic I*k*B-alfa levels were increased, in both the phosphorylated and dephosphorylated form. Ubiquitin-bound I*k*B-alfa was however reduced. These results, in agreement with the data of the total protein ubiquitination state, may be due to proteasome hyperfunction or to a defect of specific ubiquitin ligases activities, such as F-box proteins, or the recently described MIB1 [93], downregulated in this microarray experiment. As NF-*k*B signalling is deeply modulated by ubiquitination at several levels [94,95], further studies on the regulation of NF-*k*B by ubiquitination in DS may add new clues to the pathogenesis of DS.

An additional level of interest comes from recent evidence of an interplay between NF-*k*B and NFAT pathway. In fact, NF-*k*B-inducing kinase interacts with and specifically phosphorylates the C-terminal region of DSCR1 in immortalized hippocampal cells as well as in primary cortical neurons, increasing DSCR1 protein stability and blocking its proteasomal degradation [44]. This could lead to an increase in soluble and insoluble DSCR1 levels that are cytotoxic [29]. Moreover, DSCR1 overexpression stabilizes I*k*B-alfa and decreases the steady-state activity of NF-*k*B, thus inhibiting the induction of genes involved in the inflammatory response [30].

### The role of DYRK1A/DSCR1/NFAT genes in DS

The nuclear factors of activated T cells (NF-ATs) are a family of transcription factors that transduce calcium signals in the immune, cardiac, muscular, and nervous systems [96]. Like their distant relatives, the Rel family, which includes NF-*k*B, NFATs are located in the cytoplasm of resting cells and are activated for nuclear translocation [97]. Calcium signalling activates calcineurin and induces the movement of NFAT proteins into the nucleus, where they cooperate with other proteins to form complexes on DNA. As DYRK1A and DSCR1 regulate NFAT nuclear translocation, in the present study the DYRK1A/DSCR1/NFAT genes were analysed. DYRK1A at both RNA and protein levels resulted significantly overexpressed in DS. The same result was obtained for DSCR1, while a reduction of NFATc2 was observed at mRNA and protein level in DS resting cells. Reduction of NFATc2 was also observed in the nuclear fractions of DS after calcium flows stimulation. Together with the reduced NF-*k*B activity, this observation suggests the presence of a transcriptional regulation deficit in DS.

## Conclusions

Results from the present microarray analysis in human LCLs show that the UPS functioning is impaired in DS. Among genes contributing to the ubiquitin mediated proteolysis and represented on the array, a number of E2-conjugating enzymes (such as UBE2A, UBE2B, UBE2H) and E3 ligases (such as UBE3A, ITCH, SMURF2, MIB1 and some F-box proteins) resulted down-regulated. Additional bioinformatic tools such as Connectivity Map and PASS analysis add supportive evidence of the involvement of the UPS in the pathogenesis of DS. Experimental data confirm a reduction of Ub-bound proteins and an increased proteasomal acitivity. Proteasome activity may be accelerated by increased damaged or misfolded proteins and/or by chronic oxidative stress, a known biochemical feature of DS resulting from either gene dosage effect of certain chr 21 genes (i.e. SOD1, CBS, APP) or mitochondrial dysfunction [36,89,98,99].

Increased proteasome activity, along with defective ubiquitination, may lead to reduction of Ub-bound proteins, including critical regulators of cellular homeostasis such as transcriptional activators or their inhibitors. Expression analysis pointed to a down-regulation of NF-*k*B, the final actor of a complex pathway of signal transduction finely modulated by ubiquitination at several levels [94]. Interestingly, definition of crosstalk between NF-*k*B signalling pathway, ubiquitin-dependent proteolysis and the critical chr 21 gene DSCR1 is still ongoing.

Because of its central role in the cell life, the proteasome has become a target for synthetic and natural drugs for the prevention and treatment of several conditions [100-102]. An example of the medical importance of its inhibition could be the stabilization of the transcription factor NF-*k*B, required to preserve cell viability in response to environmental stress or cytotoxic agents [103-105]. Furthermore, as suggested by our findings, also the DSCR1 protein levels, overexpressed in trisomic subjects, may be regulated by the proteasome inhibition.

Recent biochemical and epidemiological studies indicate that dietary minor components, such as polyphenols, may have a role in the defense against the OS in vivo [106]. Among the different phenolic compounds, epigallocatechin-3-gallate (EGCG), the major catechin of green tea, mitigates OS and inhibits the chymotrypsin-like activity of the proteasome [107-110]. Interestingly, the over-expression of DYRK1A can also be modulated by EGCG [111] and transgenic mice overexpressing this gene and presenting cognitive impairment, rescue the cognitive phenotype after a polyphenol-based diet [112]. Our functional analyses revealed an increase of the proteasome chymotrypsin-like activity in DS subjects and the Connectivity map and PASS-assisted exploration of our microarray data suggested that some compounds with proteasome inhibitor activity could revert the biological status of DS.

In conclusion, the present work offers new perspectives to better understand the pathogenesis of DS disease and suggests a rational for innovative approaches to DS treatment.

### Availability of supporting data

Microarray data were deposited in the ArrayExpress database (http://www.ebi.ac.uk/arrayexpress/) (accession n. E-MTAB-1238).

## Abbreviations

DS: Down syndrome; chr: chromosome; LCLs: Lymphoblastoid cell lines; NFAT: Nuclear factor of activated T-cells; UPS: Ubiquitin-proteasome system; NF-kB: Nuclear factor-*k*B; FC: Fold change; FE: Fold enrichment; CV: Coefficient of variation; OS: Oxidative stress; EGCG: Epigallocatechin-3-gallate.

## Competing interests

The authors declare that they have no competing interests.

## Authors’ contributions

BG carried out most of the experiments of cell cultures, western blot, functional studies and statistical analysis; IS conceived and designed the study, participated to data interpretation and drafted the manuscript; CS performed NFATc2 western blot; AV and MC participated to sample collection and carried out some of the western blot experiments; RAV participated to data interpretation and critically revised the manuscript; PDL performed microarray experiments and statistical analysis; GA was involved in the study coordination and in the critical revision of the manuscript, giving the final approval of the version to be published. All authors read and approved the final manuscript.

## Pre-publication history

The pre-publication history for this paper can be accessed here:

http://www.biomedcentral.com/1755-8794/6/24/prepub

## Supplementary Material

Additional file 1 **A) Volcano plot of genes differentially expressed in trisomic vs control samples. B)** Clustering of genes and conditions at p-value < 0.05; **C)** Clustering of genes and conditions at p-value < 0.01 (GeneTree Algorithm). The log2 of fold change between trisomic and control samples is represented on the x-axis and the negative log of p-values from the t-test is represented on the y-axis. Up-regulated genes are represented on the right side of the horizontal axis 0-value; down-regulated gene are on the left. Red dots indicate genes that are significantly up- or down-regulated at p < 0.01. Fold-change filter in DS vs controls ≥ 1.2. Supervised hierarchical clustering of both the 4,490 and the 406 transcripts clearly distinguish between DS and control samples. Click here for file

Additional file 2 List of chr 21 transcripts, tightly regulated in both DS and control samples (CV ≤ 0.2). Click here for file

Additional file 3 Average expression ratios and Pearson’s correlation coefficient (r) between microarray and RT-PCR data. Click here for file

Additional file 4 Selection of compounds with possible therapeutic potential in DS according to the Connectivity Map and the PASSonline software. Click here for file
