# Supplementary material for: Validation of microarray data in human lymphoblasts shows a role of the ubiquitin-proteasome system and NF-kB in the pathogenesis of Down syndrome
Source: BMC Med Genomics. 2013 Jul 5;6:24. doi: 10.1186/1755-8794-6-24 (PMC3717290; doi:10.1186/1755-8794-6-24)

**Additional file 1.** A) Volcano plot of genes differentially expressed in trisomic vs control samples. B) Clustering of genes and conditions at p-value < 0.05; C) Clustering of genes and conditions at p-value < 0.01 (GeneTree Algorithm). The log2 of fold change between trisomic and control samples is represented on the x-axis and the negative log of p-values from the t-test is represented on the y-axis. Up-regulated genes are represented on the right side of the horizontal axis 0-value; down-regulated gene are on the left. Red dots indicate genes that are significantly up- or down-regulated at p< 0.01. Fold-change filter in DS vs controls > 1.2. Supervised hierarchical clustering of both the 4,490 and the 406 transcripts clearly distinguish between DS and control samples


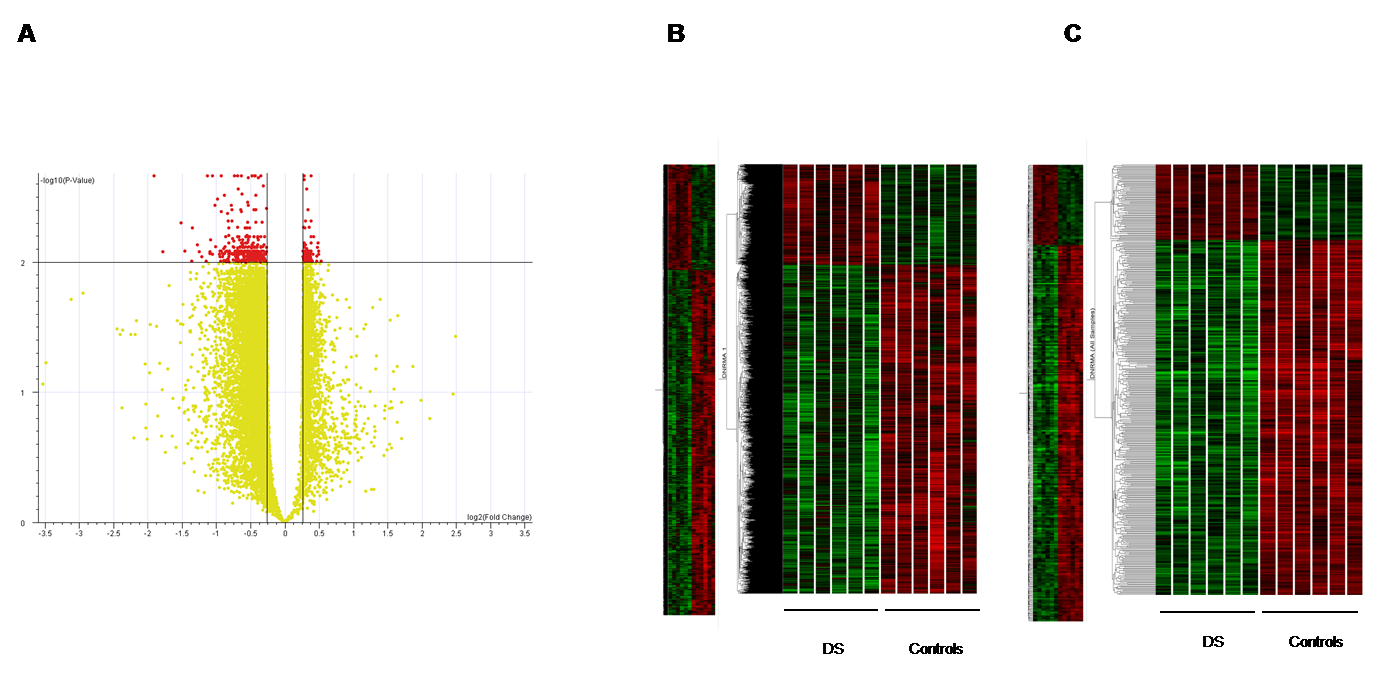

Supplement: Additional file 1 — A) Volcano plot of genes differentially expressed in trisomic vs control samples. B) Clustering of genes and conditions at p-value < 0.05; C) Clustering of genes and conditions at p-value < 0.01 (GeneTree Algorithm). The log2 of fold change between trisomic and control samples is represented on the x-axis and the negative log of p-values from the t-test is represented on the y-axis. Up-regulated genes are represented on the right side of the horizontal axis 0-value; down-regulated gene are on the left. Red dots indicate genes that are significantly up- or down-regulated at p < 0.01. Fold-change filter in DS vs controls ≥ 1.2. Supervised hierarchical clustering of both the 4,490 and the 406 transcripts clearly distinguish between DS and control samples. [file 1755-8794-6-24-S1.doc]
