# Supplementary material for: Validation of microarray data in human lymphoblasts shows a role of the ubiquitin-proteasome system and NF-kB in the pathogenesis of Down syndrome
Source: BMC Med Genomics. 2013 Jul 5;6:24. doi: 10.1186/1755-8794-6-24 (PMC3717290; doi:10.1186/1755-8794-6-24)
Supplement: Additional file 3 — Average expression ratios and Pearson’s correlation coefficient (r) between microarray and RT-PCR data. [file 1755-8794-6-24-S3.doc]

| **Additional file 3. Average expression ratios and Pearson’s correlation coefficient (r) between microarray and RT-PCR data.** | | | |
| --- | --- | --- | --- |
| **Gene** | **FC Microarray** | **RTqPCR Ratio** | **Pearson's Correlation (r)** |
| CDC27 | 0.8 | 0.79 | 0.94 |
| SMURF2 | 0.65 | 0.59 | 0.86 |
| CUL3 | 0.8 | 0.98 | 0.92 |
| ATP6 V1C1 | 0.63 | 0.58 | 0.89 |
| ATP 5O | 1.21 | 1.66 | 0.97 |
| ATP5J | 1.23 | 1.37 | 0.95 |
| NDUFV3 | 1.35 | 1.3 | 0.94 |
| U2AF1 | 1.54 | 1.3 | 0.85 |
| CBR1 | 1.3 | 2.09 | 0.93 |
| FTCD | 1.26 | 2.99 | 0.99 |
| ABCG1 | 2.57 | 5.48 | 0.92 |
