# Supplementary material for: Validation of microarray data in human lymphoblasts shows a role of the ubiquitin-proteasome system and NF-kB in the pathogenesis of Down syndrome
Source: BMC Med Genomics. 2013 Jul 5;6:24. doi: 10.1186/1755-8794-6-24 (PMC3717290; doi:10.1186/1755-8794-6-24)
Supplement: Additional file 4 — Selection of compounds with possible therapeutic potential in DS according to the Connectivity Map and the PASSonline software. [file 1755-8794-6-24-S4.doc]

**Additional file 4. Selection of compounds with possible therapeutic potential in DS according to the Connectivity Map and the PASSonline software.**

| **Cmap name** | **Cmap enrichment score** | **Cmap**  **p-value** | **Predicted activities* possibly relevant to DS treatment** |
| --- | --- | --- | --- |
| Adiphenine | -0.847 | 0.0002 | Antineurotoxic  Antiparkinsonian  Proteasome ATPase inhibitor  Ubiquitin thiolesterase inhibitor  Proteasome endopeptidase complex inhibitor activity |
| Thioperamide | -0.808 | 0.0006 | GABA release inducer  [H3 receptor antagonist](http://en.wikipedia.org/wiki/H3_antagonist) |
| Vigabatrin | -0.925 | 0.0007 | Proteasome ATPase inhibitor  GABA transaminase inhibitor  Apoptosis antagonist |
| 3-acetamidocoumarin | -0.856 | 0.0007 | Nerve growth factor agonist  Apoptosis antagonist |
| Biperiden | -0.782 | 0.0009 | Antiparkinsonian  Antineurotoxic  Nerve growth factor agonist |
| Viomycin | -0.851 | 0.0009 | Protein-synthesizing GTPase inhibitor |
| Lisuride | -0.779 | 0.0009 | Antiparkinsonian  Apoptosis antagonist  Dopamine antagonist  GABA receptor antagonist |
| Prestwick-692 | -0.845 | 0.0010 | Not available on PASS |
| Eticlopride | -0.838 | 0.0012 | Proteasome ATPase inhibitor  Apoptosis antagonist  Neuroprotector  Dopamine antagonist |
| Prestwick-1103 | -0.832 | 0.0014 | Not available on PASS |
| Isoflupredone | -0.900 | 0.0019 | Antiinflammatory steroid  Phosphatase inhibitor |
| Oxybenzone | -0.811 | 0.0025 | Free radical scavenger  Cytoprotectant  Neurotrophic factor enhancer  Nerve growth factor agonist  Ligase inhibitor |
| Podophyllotoxin | -0.802 | 0.0030 | Phosphatase inhibitor  GABA receptor antagonist  Topoisomerase I inhibitor  Neurotrophic factor enhancer  Cytoprotectant |
| Hexetidine | -0.801 | 0.0030 | Apoptosis antagonist  NMDA receptor glycine site agonist |
| Prestwick-691 | -0.884 | 0.0031 | Not available on PASS |
| Heptaminol | -0.706 | 0.0048 | Proteasome endopeptidase complex inhibitor  Superoxide dismutase inhibitor  Apoptosis antagonist  NMDA receptor glycine site agonist |
| Timolol | -0.778 | 0.0050 | Phosphatase inhibitor  Beta adrenoreceptor antagonist |

The table displays compounds with enrichment score <-0.7 ordered by p-value, according to Connectivity map.

* The predicted activity is based on PASSonline software (Prediction of Activity Spectra for Substances). Some of the predicted activities are shared by more than one molecule. The possible activities relevant to DS were selected based on the present knowledge on DS pathogenesis. Only compounds with Pa>0.5 are reported in the table. The entire list of the predicted activities for each compound is found at <http://www.pharmaexpert.ru/passonline/index.php>.
